# Genotyping analysis and ^18^FDG uptake in breast cancer patients: a preliminary research

**DOI:** 10.1186/1756-9966-32-23

**Published:** 2013-04-30

**Authors:** Valentina Bravatà, Alessandro Stefano, Francesco P Cammarata, Luigi Minafra, Giorgio Russo, Stefania Nicolosi, Sabina Pulizzi, Cecilia Gelfi, Maria C Gilardi, Cristina Messa

**Affiliations:** 1 IBFM CNR - LATO, Cefalù, PA, Italy; 2 US of Nuclear Medicine, Fondazione Istituto “San Raffaele - G. Giglio”, Cefalù, PA, Italy; 3 Department of Biomedical Sciences for Health, University of Milan, Milan, Italy; 4 Nuclear Medicine, San Raffaele Scientific Institute, Milan, Italy; 5 Department of Health Sciences, Tecnomed Foundation, University of Milano-Bicocca, Milan, Italy; 6 Nuclear Medicine Center, San Gerardo Hospital, Monza, Italy

**Keywords:** Breast cancer, Single nucleotide polymorphisms, PET-CT, SUVmax, SUVpvc

## Abstract

**Background:**

Diagnostic imaging plays a relevant role in the care of patients with breast cancer (BC). Positron Emission Tomography (PET) with 18F-fluoro-2-deoxy-D-glucose (FDG) has been widely proven to be a clinical tool suitable for BC detection and staging in which the glucose analog supplies metabolic information about the tumor. A limited number of studies, sometimes controversial, describe possible associations between FDG uptake and single nucleotide polymorphisms (SNPs). For this reason this field has to be explored and clarified. We investigated the association of SNPs in GLUT1, HIF-1a, EPAS1, APEX1, VEGFA and MTHFR genes with the FDG uptake in BC.

**Methods:**

In 26 caucasian individuals with primary BC, whole-body PET-CT scans were obtained and quantitative analysis was performed by calculating the maximum Standardized Uptake Value normalized to body-weight (SUVmax) and the mean SUV normalized to body-weight corrected for partial volume effect (SUVpvc). Human Gene Mutation Database and dbSNP Short Genetic Variations database were used to analyze gene regions containing the selected SNPs. Patient genotypes were obtained using Sanger DNA sequencing analysis performed by Capillary Electrophoresis.

**Results:**

BC patients were genotyped for the following nine SNPs: GLUT1: rs841853 and rs710218; HIF-1a: rs11549465 and rs11549467; EPAS1: rs137853037 and rs137853036; APEX1: rs1130409; VEGFA: rs3025039 and MTHFR: rs1801133. In this work correlations between the nine potentially useful polymorphisms selected and previously suggested with tracer uptake (using both SUVmax and SUVpvc) were not found.

**Conclusions:**

The possible functional influence of specific SNPs on FDG uptake needs further studies in human cancer. In summary, this is the first pilot study, to our knowledge, which investigates the association between a large panel of SNPs and FDG uptake specifically in BC patients. This work represents a multidisciplinary and translational medicine approach to study BC where, the possible correlation between SNPs and tracer uptake, may be considered to improve personalized cancer treatment and care.

## Background

Breast cancer (BC) is the leading cause of cancer-related death in women world-wide [1] and presents distinct subtypes associated with different clinical outcomes. Understanding this heterogeneity represents a key factor for the development of targeted preventive and therapeutic interventions [2-4]. Upon cancer disease occurrence, survival outcomes seem to be dependent not only on the histological type but also on the intensity of lesion measured by ^18^F-fluoro-2-deoxy-D-glucose Positron Emission Tomography (FDG PET) uptake [5]. FDG PET is a non-invasive diagnostic and prognostic tool that assess tumour metabolism and it is used for treatment planning and the evaluation of therapy response [6]. To improve this technique, it is necessary to discover deregulated tumour-specific markers that may serve as molecular targets for the imaging of cancer [7].

The completion of nine large genome-wide association studies [8,9] introduced single-nucleotide polymorphisms (SNPs) as risk factors for BC disease [10]. Despite considerable progress, their commercial exploitation in clinical applications remains controversial [11,12]. In addition, the potential functional influence of specific SNPs on tracer PET uptake needs further investigations in human cancer diseases. Indeed, the first study demonstrating an association between a human SNP (rs3025039 of the Vascular Endothelial Growth Factor A, abbreviated as VEGFA) and FDG uptake in BC, has included a restricted number of 37 ductal BC patients without metastases [13].

Although, the possible correlation between gene polymorphisms and FDG uptake is considered an innovative and interesting example of translational medicine approach, where information from multiple sources are combined aiming to a more personalized care, the number of scientific papers is still limited [13-18]. Nowadays, candidate targets used for these studies are polymorphisms in the GLUcose Transporter 1 gene (GLUT1 also known as SLC2A1) and the following three hypoxia-related genes: Hypoxia-Inducible Factor 1alpha (HIF-1a), VEGFA and apurinic/apyrimidinic APEX nuclease 1 (APEX1) [13-18].

GLUT family members are often over-expressed in most human malignancies [19] and are involved in tumour initiation and progression. However, they are already present in the respective non-cancerous tissue of origin. The class I transporters (GLUT1), and to a much less extent GLUT3, are the most frequently over-expressed genes in cancer cells. Their over-expression positively correlates with several adverse tumour characteristics and PET uptake in BC [20] and various other malignancies [21-23]. Regarding the role of GLUT1 on PET imaging, only two authors have shown that rs841853and rs710218 GLUT1 SNPs influence tracer PET uptake [14,15]. These two SNPs were considered to be able to determine variations on the behaviour of the glucose transporter in various human diseases, such as diabetic nephropathy and clear-cell renal carcinoma [24,25], where a high significant allele frequency in the population investigated was found, suggesting their potential clinical application. The rs841853 SNP is located in a non-protein coding region (intron 2 of the GLUT1 gene) and seems to have a role in recruiting glucose over the membrane, accelerating growth cell rate. The rs710218 SNP is positioned in the promoter region of the GLUT1 gene adjacent to a putative HIF-1a binding site [26]. HIF-1a controls oxygen delivery and metabolic adaptation to hypoxia via angiogenesis and glycolysis, respectively and it also regulates, under hypoxic conditions, the expression of genes, like the GLUT1 gene. Numerous clinico-pathological studies have demonstrated that unlike mature normal tissues, 40%-80% of human carcinomas, like melanomas, sarcomas, head and neck, lung, breast and endometrial cancers contain elevated cytoplasmic and nuclear HIF-1a levels [27-29]. In addition, Fu XS. et al. and Koukourakis MI. et al. showed that HIF-1a gene polymorphisms, such as rs11549465 and rs11549467, affect its expression [30,31]. These SNPs seem to be also related with FDG uptake as described by Kim SJ. and co-workers [15].

Hypoxia-inducible factor 2 alpha (HIF-2a), also known as endothelial PAS domain protein 1 (EPAS1), is another member of the hypoxia-inducible factor family and shares many similarities with HIF-1a [32,33]. However several molecular, biochemical, and physiological studies have established that HIF-1a and HIF-2a are not redundant but have distinct functions [34]. To understand the possible relationship of EPAS1 and the abovementioned HIF-1a SNPs to FDG uptake, we analyzed the only two EPAS1 missense mutations (rs137853037 and rs137853036) with probable pathogenicity as described in the dbSNP Short Genetic Variations database and in the Human Gene Mutation Database where a collection of known gene lesions responsible for human inherited diseases is found.

APEX1, a DNA base excision repair enzyme, has also a role in transcriptional activation of HIF-1 and the hypoxia inducible factor-like factor (HLF). APEX1 polymorphisms have been the object of studies about in several types of cancer including colorectal, breast and non-small cell lung cancer (NSCLC) in order to evaluate their role in cancer susceptibility, development and response to radiotherapy [15,35]. Interestingly, in NSCLC patients with the APEX1 rs1130409 TT genotype an association, not fully clarified yet, between the abovementioned rs710218 GLUT1 SNP and FDG uptake was shown [15].

Overall, all previous studies have investigated SNPs of a limited number of genes. Furthermore, the type of cancer tissue varies, rendering difficult the evaluation of their real impact on FDG PET uptake in specific cancer types. To our knowledge, no studies have examined the simultaneous presence and role of these specific polymorphisms in BC patients. Therefore, the purpose of this preliminary research was to highlight possible associations between the abovementioned SNPs of the GLUT1, HIF-1a, EPAS1, APEX1 and VEGFA genes and the FDG uptake, in order to identify a large panel of SNPs, for imaging analysis that will allow a more personalized treatment program.

## Methods

### Patients

Thirty-three caucasian individuals with primary BC were enrolled for a multidisciplinary project named “*Tissue characterization in primary BC: correlation with FDG-PET uptake and with choline peak by proton nuclear MR spectroscopy”.* Inclusion criteria for genotyping analysis were: patients candidated for surgery of invasive BC with a tumour size of at least 2 cm, as measured by mammography and breast ultrasonography and not treated with primary chemotherapy. Twenty-six BC patients were finally selected for genotyping analysis using the abovementioned inclusion criteria. The study and the consent procedure were performed according to the Helsinki declaration and were approved by the Ethical Committee (EC) of HSR G. Giglio hospital, Cefalù-Italy.

### FDG PET-CT

Before surgical resection of primitive BC, all patients underwent FDG PET-CT studies. The patients were fasted for twelve hours before performing PET-CT scan, and were injected intravenously with FDG (37MBq/10 kg). Patients with a blood glucose level greater than 150 mg/dl were not included in the study. The weight of each patient was measured the day of the PET-CT study. Actual injected and residual radioactivity were measured by the dose measurement system. PET-CT acquisition started 50 min after radiotracer injection and images were acquired from the top of the skull to the middle of the thigh with the arms raised. Whole-body PET-CT scans were obtained using a Discovery STE scanner (General Electric Medical Systems), installed at the Nuclear Medicine Department of LATO-HSR (Cefalù, Italy). The system is a three-dimensional BGO 47 slice PET scanner combined with an helical 8 slice CT scanner. The PET-CT oncological protocol included a low dose CT scan and a 3D PET whole body scan (2.5 min/bed position). Patients breathed normally during the PET and CT exams. PET images were reconstructed by a 3D ordered subset expectation maximization algorithm (OSEM, 28 subsets, 2 iterations, 5.14 mm Gaussian post-smoothing) with corrections for random, scatter and attenuation incorporated into the iterative process.

### Quantitative PET measurements

Quantitative analysis was performed calculating, for each breast lesion, the maximum Standardized Uptake Value (SUVmax) and the mean SUV (SUVpvc) normalized to body-weight. Partial volume effect correction (PVC) was performed to compensate spill in (signal from background region that goes inside the lesion) and spill out (signal from the lesion that goes into background region) effects in the SUVpvc [36,37]. Since the SUVmax is the uptake index least affected by partial volume effect no correction was applied.

Briefly, the PVC method is based on recovery coefficient (RC) curves obtained from NEMA 2001 IQ phantom (equipped with six spheres of different sizes - from 10 mm to 37 mm- to account for size effect) as a function of PET measured metabolic volume and of PET measured sphere-to-background ratio [38]. The metabolic volume was calculated as the 60% isocontour of the maximum pixel intensity automatically drawn on the PET lesion. The radioactivity concentration in the lesion was measured as the average radioactivity concentration within the metabolic volume. The background radioactivity concentration was obtained as the average of four circular ROIs positioned over the background around the lesion. To apply the PVC correction method, PET measured metabolic volumes and lesion-to-background ratios were considered within the following ranges of RC curves: measured diameters (derived from metabolic volume) from 0 to 4 cm and lesion-to-background ratios from 2 to 30.

### BC patients genotyping

In this study BC patients were genotyped, by sequencing analysis, for the following polymorphisms: GLUT1 rs841853 (also known as *Xba*I because is a restriction fragment length polymorphism-RFLP) and rs710218 SNPs (also known as *Hpy*CH4V); HIF-1a rs11549465 and rs11549467 SNPs; EPAS1 rs137853037 and rs137853036 SNPs; APEX1 rs1130409 SNP; VEGFA rs3025039 and MTHFR rs1801133 SNP.

Human Gene Mutation Database [39] and dbSNP Short Genetic Variations database [40] were used to analyze gene regions containing the selected SNPs. Genomic DNA was extracted from peripheral blood using QIAamp DNA blood mini kit, according to the manufacturer’s specifications (Qiagen). After quality and quantity analysis, genomic DNA was PCR amplified using primers designed by the Primer3 software [41] and listed in Table 1. PCR reactions were performed with 50 ng of genomic DNA in a total volume of 50 μL containing 1X PCR Gold Buffer, 1,5 mM di MgCl2, 200 μM dNTPs, 200 nM of forward and reverse primer mix, 1.25 U of AmpliTaq Gold DNA Polymerase (Applied Biosystems). The thermal cycle profile employed a 5-min denaturing step at 94°C, followed by 35 cycles at 94°C for 45 sec, 59°C for 45 sec, 72°C for 45 sec and a final extension step of 5 min at 72°C.

**Table 1:** Primers sequence used for genotyping analysis

**Target gene polymorphism (rs number)**	**Forward primer 5′ > 3′**	**Reverse primer 5′ > 3′**	**Template size (base pairs)**
**GLUT1 _Xba I G > T**	gtgcaacccatgagctaacaa	aacccagcactctgtagcc	305
**(rs841853)**			
**GLUT1 _HpyCH4V −2841 A > T**	tgagaatggccttccctcaat	tctgccttactcagcccatg	336
**(rs710218)**			
**HIF1a Pro582Ser**	cccaatggatgatgacttcc	tctgtttggtgaggctgtcc	316
**(rs11549465)**			
**HIF1a Ala588Thr**	cccaatggatgatgacttcc	tctgtttggtgaggctgtcc	316
**(rs11549467)**			
**EPAS1 Met535Val**	tgacacagccaagtctgagg	ggctctcaacaagccacttc	902
**(rs137853037)**			
**EPAS1 Gly537Arg**	tgacacagccaagtctgagg	ggctctcaacaagccacttc	902
**(rs137853036)**			
**APEX1 Asp148Glu**	gccagtgcccactcaaagtt	cttgcgaaaggcttcatccc	176
**(rs1130409)**			
**VEGFA +936 C > T**	ctcctcacttggccctaacc	gggtgggtgtgtctacagga	414
**(rs3025039)**			
**MTHFR Ala222Val**	tttctatggccaccaagtgcag	gacactgttgctgggttttgg	716
(rs1801133)			

Quality and quantity of PCR products were assessed on the Bioanalyzer instrument (Agilent Technologies) and were purified using QIAquick PCR purification kit (Qiagen), according to the manufacturer’s specifications. To perform DNA sequencing, purified amplicons were labelled with BigDye Terminator v3.1 Cycle Sequencing Kit following the manufacturer’s standard protocol (Applied Biosystems). The thermal cycle profile employed a 1 min denaturing step at 96°C, followed by 25 cycles at 96°C for 10 sec, 54°C for 5 sec, 60°C for 3 min. Labelled samples were purified with X-terminator purification kit according to manufacturer’s standard protocol and loaded in 3500-Dx Genetic Analyzer (Applied Biosystems) for separation by capillary electrophoresis. Electropherograms and sequence files were analyzed using Sequencing Analysis and SeqScape softwares (Applied Biosystems).

### Statistical analysis

The association between the SUVmax and SUVpvc values and genetic factors was analyzed using GraphPad InStat software version 3.05, San Diego California USA. Mann Whitney-*U* test and Fisher’s exact test were performed. Differences in groups for the medians SUVmax and SUVpvc values were tested. Differences were considered significant when *p* value was less than or equal to 0.05.

## Results

### Patients

The average age of 26 selected BC patients for genotyping analysis was 56.9 y (age range, 36–88 y; SD, 15.6 y).

### FDG PET-CT & quantitative PET measurements

SUVmax and SUVpvc values are shown in Table 2. The average of SUVmax was 7.67 ± 4.01 (range: 1.95-17.65; 95% confidence interval (C.I.) 6.05-9.29). The average of SUVpvc was 7.58 ± 3.88 (range: 2.64-19.15;; 95% C.I. 6.02-9.15), the mean sphere-equivalent diameter of PET measured metabolic volume was 1.39 ± 0.44 cm (range: 0.8-2.55; 95% C.I. 1.21-1.56) and the average PET measured lesion-to-background ratio was 12.12 ± 5.65 (range: 1.92-25.79; 95% C.I. 9.84-14.40). In all cases the lesions had a measured sphere-equivalent diameter and a measured lesion-to-background ratio within the range of the RC curves. PET-TC images will be available in confidence with the radiology reader upon request.

**Table 2:** SUVmax and SUVpvc values

**ID patient**	**SUVmax**	**SUVpvc**
Pz1	3,93	3,62
Pz2	10,91	9,95
Pz3	5,68	5,83
Pz4	5,81	5,76
Pz5	8,62	7,19
Pz6	11,74	10,94
Pz7	4,08	4,35
Pz8	5,34	5,83
Pz9	9,25	8,66
Pz10	11,97	11,58
Pz11	12,85	10,29
Pz12	4,95	4,25
Pz13	10,59	9,89
Pz14	8,03	8,36
Pz15	14,61	19,15
Pz16	5,25	5,89
Pz17	4,12	4,01
Pz18	6,6	7,39
Pz19	2,79	3,22
Pz20	5,27	6,32
Pz21	9,23	7,81
Pz22	17,65	15,15
Pz23	2,82	3,13
Pz24	4,85	5,64
Pz25	1,95	2,64
Pz26	10,47	10,24

### BC patients mutation analysis of the eight SNPs panel

BC patients, were genotyped for the eight SNPs previously introduced (GLUT1: rs841853 and rs710218; HIF-1a: rs11549465 and rs11549467; EPAS1: rs137853037 and rs137853036; APEX1: rs1130409; VEGFA: rs3025039). Allele frequencies and the percentages of the three possible genotypes for each SNP were calculated. Deviations of Hardy-Weinberg equilibrium were not observed for all SNPs except for the rs3025039 VEGFA polymorphism (Table 3).

**Table 3:** SNPs analysis results

**SNP**	**n = 26**	**%**	**Allele frequencies**	**Hardy-Weinberg equilibrium**
**GLUT1 (rs841853)**				
GG	7	26,9	G = 0,442	p =0,13
TG	9	34,6	T = 0,558	
TT	10	38,5		
**GLUT1 (rs710218)**				
AA	15	57,7	A = 0,788	p =0,17
AT	11	42,3	T = 0,212	
TT	0	0		
**HIF1a (rs11549465)**				
CC	21	80,7	C = 0,904	p =0,59
CT	5	19,3	T = 0,096	
TT	0	0		
**HIF1a (rs11549467)**				
GG	25	96,2	G = 0,981	p =0,92
GA	1	3,8	A = 0,019	
AA	0	0		
**EPAS1 (rs137853037)**				
AA	26	100	A = 1	NA
AG	0	0	G = 0	
GG	0	0		
**EPAS1 (rs137853036)**				
GG	26	100	G = 1	NA
GA	0	0	A = 0	
AA	0	0		
**APEX1 (rs1130409)**				
TT	9	34,6	T = 0,596	p =0,84
TG	13	50	G = 0,404	
GG	4	15,4		
**VEGFA (rs3025039)**				
CC	20	76,9	C = 0,846	p =0,04
CT	4	15,4	T = 0,154	
TT	2	7,7		
**MTHFR (rs1801133)**				
CC	6	23,1	C = 0,442	p =0,47
CT	11	42,3	T = 0,558	
TT	9	34,6		

*NA*, not available.

### Association between SNPs and SUV values

In order to evaluate a potential association between the SNPs and the PET tracer uptake, SUVmax and SUVpvc values were used. Patients were divided in subgroups according to SNP genotype and a Mann-Whittney statistical test was performed to evaluate the differences in SUVmax and SUVpvc levels. Unfortunately, the genotype sample size for HIF-1a: rs11549467 and EPAS1: rs137853037 and rs137853036 SNPs was insufficient to apply a statistical analysis (Table 3). No genotype of the selected SNPs showed any significant association with PET tracer uptake (Table 4).

**Table 4:** Association between genotype and SUVmax and SUVpvc values in BC patients

**SNP**		**SUVmax**	**SUVmax**		**SUVpvc**	**SUVpvc**
			p -values*			p -values*
**SLC2A1 (rs841853)**						
GG	GG	5,771 ± 2,475	0,1882	GG	5,619 ± 2,309	0,1067
TG	GT + TT	8,366 ± 4,293		GT + TT	8,303 ± 4,135	
TT						
**SLC2A1 (rs710218)**						
AA	AA	7,497 ± 4,032	0,7988	AA	7,074 ± 3,200	0,6591
AT	AT + TT	7,901 ± 4,175		AT + TT	8,271 ± 4,735	
TT						
**HIF1a (rs11549465)**						
CC	CC	7,387 ± 3,850	0,4861	CC	7,214 ± 3,237	0,6724
CT	CT + TT	8,848 ± 4,948		CT + TT	9,118 ± 6,172	
TT						
**APEX1 (rs1130409)**						
TT	TT	6,607 ± 3,360	0,3388	TT	6,412 ± 3,051	0,3187
TG	TG + GG	8,229 ± 4,310		TT + GG	8,119 ± 4,208	
GG						
**VEGFA (rs3025039)**						
CC	CC	8,107 ± 4,178	0,3875	CC	7,997 ± 4,038	0,3302
CT	CT + TT	6,205 ± 3,307		CT + TT	6,193 ± 3,218	
TT						
**MTHFR (rs1801133)**						
CC	CC	8,415 ± 5,367	0,9292	CC	7,687 ± 4,390	0,9764
CT	CT + TT	7,444 ± 3,661		CT + TT	7,549 ± 3,840	
TT						

* Mann Whitney-*U* Test.

We also classified the patients into subgroups according to their SUV values (subgroup with high SUV values versus low SUV values one, for both SUVmax and SUVpvc). A Fisher’s exact analysis confirmed that no significant association between PET tracer uptake and specific SNP profiles exists.

Kim SJ. and colleagues have shown that the GLUT1 rs710218 polymorphism is significantly associated with SUVmax in combination with APEX1 rs1130409 SNP in NSCLC disease [15]. To investigate its putative role in FDG uptake in BC, we studied the association between the GLUT1 rs710218 SNP and SUVmax and SUVpvc in patients classified according the APEX1 rs1130409 genotype. The levels of SUVmax and SUVpvc were similar because *p* value was greater than 0.05 in all GLUT1 rs710218 genotype groups regardless the APEX1 rs1130409 genotype (Table 5).

**Table 5:** Association between the rs710218 GLUT1 SNP and SUVmax and SUVpvc values in BC patients according to APEX1 rs1130409 genotype

**SNP**	**Genotype**	**GLUT1 rs710218 genotypes**	**SUVmax**	SUVmax p -values*	**SUVpvc**	SUVpvc p -values*
**APEX1 rs1130409**	**TT**	AA	6,735 ± 1,859	0,7302	6,408 ± 1,771	0,9048
	**(n = 9)**	AT + TT	6,504 ± 4,467		6,416 ± 4,034	
	**TG**	AA	7,048 ± 4,763	0,3301	6,931 ± 3,890	0,414
	**(n = 13)**	AT + TT	8,525 ± 3,328		8,480 ± 2,413	
	**GG**	AA	11,040 ± 2,560	>0,9999	9,050 ± 1,754	>0,9999
	**(n = 4)**	AT + TT	10,145 ± 6,314		12,490 ± 9,419	

*Mann Whitney-*U* Test.

### MTHFR SNPs and FDG uptake association analysis

Considering that thromboembolism is a major complication in patients with BC, accompanied also by significant morbidity and mortality [42], we decided to investigate the role of thromboembolism-related SNPs in PET tracer uptake. The rs1801133 and rs181131 SNPs of the 5,10-methylenetetrahydrofolate reductase (MTHFR) gene, encoding for a key enzyme in the folate metabolism pathway, have been associated with reduced enzyme activity and hyperhomocysteinemia related with thromboembolic events [43] and affect chemosensitivity of tumour cells. In addition, Jakubowska A. and co-workers found that the rs1801133 MTHFR SNP is associated with an increased risk for breast and ovarian cancer [44,45]. MTHFR rs1801133 allele frequencies and the percentages of the three possible genotypes were calculated and deviations of Hardy-Weinberg equilibrium were not observed [46]. No genotype of rs1801133 showed any significant association with PET tracer uptake, as revealed both by Mann-Whittney and Fisher’s exact statistical analysis because *p* value was greater than 0.05 (Table 4).

## Discussion

Today, a very limited number of reports describe possible associations between FDG uptake and SNPs, rendering this field poorly explored and clarified [13-18]. Our study investigated the possible simultaneous association between polymorphisms in GLUT1, HIF-1a, EPAS1, APEX1,VEGFA and MTHFR genes and the FDG-PET uptake. To our knowledge, this is the first work that evaluates the collective impact of the abovementioned SNPs on PET tracer uptake in BC patients. FDG uptake, expressed in terms of SUVmax or SUVpvc, is largely dependent on glucose metabolism. High values are associated with reduced overall survival in cancer patients [41].

GLUT1 is the primary transporter of glucose metabolism and its over-expression has an important role in the survival and rapid growth of cancer cells. The rs841853 polymorphism of GLUT1 is located on the second intron of the gene and as suggested by Kim SJ et al. [15], no change would be expected in the GLUT1 protein sequence and expression. However, the GG genotype, which occurs in about 52% of the European population (data derived by dbSNP Short Genetic Variations database) seems to be related to FDG uptake in BC patients [14]. In our work, although we did not observe deviation from the Hardy–Weinberg equilibrium, we did not find the association between this SNP and the FDG tumour uptake in BC.

The promoter region of the GLUT1 gene harbours another SNP, rs710218 (named also SLC2A1 *Hpy*CH4V), positioned 400 bp upstream of a putative HIF-1a binding site. Its close proximity to the hypoxia response elements (HRE) may modify the binding affinity of HIF-1 and thus alter the efficiency of the promoter and expression of GLUT1 [24]. In our study, the allele frequencies of rs710218 SNP did not differ significantly from those available in NCBI dbSNP database and no association between this genetic alteration and SUVmax or SUVpvc was found in BC patients, confirming similar data recently obtained in NSCLC [15]. No significant association with FDG uptake exists also when we examined this SNP in combination with the APEX1 rs1130409 genotypes.

APEX1 promotes transcriptional activation of HIF-1 and its reduced levels are related to a decrease in tumour volume and FDG uptake, suggesting that it affects glucose metabolism and cellular proliferation [41]. Homozygosity (TT genotype) for the rs1130409 APEX1 SNP was significantly associated with a poor overall cancer survival [15]. Here, this genotype was not significantly associated with SUV, compared with the GG/TG genotypes, as previously shown by by Kim SJ et al. [15].

HIF1a itself has an SNP (rs11549465) that we studied for possible association with FDG uptake. However, we observed no association in BC disease, in agreement with data previously obtained in NSCLC [15].

VEGFA rs3025039 polymorphism has been related with BC risk and a C > T polymorphism at position 936 in the 3’ untranslated region of the VEGFA gene has been associated with VEGF plasma levels. Specifically, the T-variant is linked to lower VEGF level and associated with increased BC risk [13] and worse outcome [17] compared to the wildtype allele. Wolf G. and co-workers [13] suggested a potential role of this VEGFA polymorphism on the variability of FDG uptake in tumour tissue. However, our study and data reported by Lorenzen S. et al. [17] do not confirm this association.

The MTHFR rs1801133 SNP is highly represented in the Caucasian population [46] and it is related to increased BC risk [36-38]. Nevertheless, its role in PET has not been studied yet. Here, we evaluated its importance in FDG uptake, for the first time, finding no associations. Considering its great importance in BC, we still believe that additional studies are needed to clarify its relevance.

Unfortunately, the genotype distributions for the remaining HIF1a: rs11549467, EPAS1: rs137853037 and rs137853036 SNPs did not allow us to evaluate their possible association with SUV.

The possible association between FDG uptake and SNPs is described by a limited number of studies, due to the need for multidisciplinary team and expertise. Moreover, this research field is characterized by controversial reports.

Moreover a strong variability of FDG-PET uptake on BC tissue has been reported [13], but the reason for this variability is not fully understood and may involve various cellular processes and risk factors such as genetic predisposition. Overall, our analysis succeeded to reproduce some previous findings, while we failed to confirm others, which still need to be further investigated. These discrepancies can be explained by the shortage of patients assayed both in our work and previous studies [13-15].

In addition these works looked at different groups of people from various European countries. Therefore it is harder to compare findings and to draw conclusions: the geographical and racial/ethnic distribution of an allele and associated genotypes are considered extremely important to fully understand the risk and the development of treatments related to gene polymorphisms (sometimes the allele frequency varies from region to region in the same country) [47].

Concluding, none of the reported analyses included functional evaluation of SNPs in FDG PET uptake. In our work, the potentially useful polymorphisms were not found associated with FDG uptake, using both SUVmax and SUVpvc.

Taking into consideration the clinical impact of a significant association between genetic alterations and PET-CT could have in BC treatment and since current knowledge is limited, additional and larger studies are required to assess the importance of these genotypic variants in the phenotypes or biological functions. Additionally, we cannot exclude the possibility that unknown or known SNPs, not investigated yet, in the same genes could have an important role.

## Conclusions

This is the first report to our knowledge investigating the association between a large panel of SNPs genotypes and FDG uptake in BC patients. In this work we shown that none of the nine potentially useful polymorphisms selected and previously suggested by other authors were statistically correlated with FDG PET-CT tracer uptake (using both SUVmax and SUVpvc). The possible functional influence of specific SNPs on FDG uptake needs further studies in human cancer. Concluding, this work represents a multidisciplinary and translational medicine approach to study BC where the possible correlation between gene polymorphisms and tracer uptake may be considered to improve personalized cancer treatment and care.

## Competing interests

The authors declare that they have no competing interests.

## Authors’ contributions

All authors participated to the conception, design, interpretation, elaboration of the findings of the study, drafting and revising the final elaborate. In particular, Dr. VB designed the study, wrote the paper and with Dr. FPC and Dr. LM performed patients genotyping experiments. Dr. SP selected and enrolled the patients and performed FDG PET-CT studies. Dr. AS performed quantitative PET measurements and with Dr. GR and Dr. SN analysed data. Prof. CG, Prof. MCG and Prof. CM participated in the elaboration of the findings of the study, drafting and revising the final elaborate. All authors read and approved the final content of the manuscript.
